# A 10-year Metocean dataset for Laguna Madre, Texas, including for the Study of Extreme Cold Events

**DOI:** 10.1016/j.dib.2023.109828

**Published:** 2023-11-19

**Authors:** Miranda C. White, Marina Vicens-Miquel, Philippe Tissot, Evan Krell

**Affiliations:** a Texas A&M University-Corpus Christi: Physical and Environmental Sciences Department, 6300 Ocean Drive, Corpus Christi, TX 78412, United States; b Texas A&M University-Corpus Christi: Computer Science Department, 6300 Ocean Drive, Corpus Christi, TX 78412, United States; c Texas A&M University-Corpus Christi: Conrad Blucher Institute, 6300 Ocean Drive, Corpus Christi, TX 78412, United States; d NSF AI Institute for Research on Trustworthy AI in Weather, Climate, and Coastal Oceanography (AI2ES), 550 Parrington Oval, Norman, OK 73019, United States

**Keywords:** Coastal system science dataset, Machine learning applications, Hypothermic stunning, Laguna Madre, Environmental data imputation, Coastal oceanography, Texas coast

## Abstract

Coastal observations along the Texas coast are valuable for many stakeholders in diverse domains. However, the management of the collected data has been limited, creating gaps in hydrological and atmospheric datasets. Among these, water and air temperature measurements are particularly crucial for water temperature predictions, especially during freeze events. These events can pose a serious threat to endangered sea turtles and economically valuable fish, which can succumb to hypothermic stunning, making them vulnerable to cold-related illness or death. Reliable and complete water and air temperature measurements are needed to provide accurate predictions of when cold-stunning events occur. To address these concerns, the focus of this paper is to describe the method used to create a complete 10-year dataset that is representative of the upper Laguna Madre, TX using multiple stations and various gap-filling methods. The raw datasets consist of a decade's worth of air and water temperature measurements within the Upper Laguna Madre from 2012 to 2022 extracted from the archives of the Texas Coastal Ocean Observation Network and the National Park Service. Large portions of data from the multiple stations were missing from the raw datasets, therefore a systematic gap-filling approach was designed and applied to create a near-continuous dataset. The proposed imputation method consists of three steps, starting with a short gap interpolation method, followed by a long gap-filling process using nearby stations, and finalized by a second short gap interpolation method. This systematic data imputation approach was evaluated by creating random artificial gaps within the original datasets, filling them using the proposed data imputation method, and assessing the viability of the proposed methods using various performance metrics. The evaluation results help to ensure the reliability of the newly imputed dataset and the effectiveness of the data imputation method. The newly created dataset is a valuable resource that transcends the local cold-stunning issue, offering viable utility for analyzing temporal variability of air and water temperatures, exploring temperature interdependencies, reducing forecasting uncertainties, and refining natural resource and weather advisory decision-making processes. The cleaned dataset with minimal gaps (<2%) is ready and convenient for artificial intelligence and machine learning applications.

Specifications Table

**Table:** 

**Subject**	Earth and Planetary Science
**Specific subject area**	Coastal Oceanography and Applied Machine Learning
**Data format**	Raw and Filtered
**Type of data**	Table
**Data collection**	Air and water temperature data were acquired from the records of the Texas Coastal Ocean Observation Network (TCOON) and the National Park Service (NPS). All data collected by TCOON follow the National Ocean Service standards including instrumentation, data collection procedures, periodic inspections and maintenance, and metadata collection.
**Data source location**	The data was collected through TCOON and NPS sources. Air temperature measurements utilized TCOON stations of Packery Channel (27° 38′ 4″ N, 97° 14′ 13″ W), Baffin Bay (27° 17′ 49″ N, 97° 24′ 17″ W), while water temperatures utilized TCOON South Bird Island station (27° 29′ 4″ N, 97° 19′ 5″ W) and NPS South Bird Island station (same location as TCOON station). The data was stored in the lighthouse database of the Conrad Blucher Institute at Texas A&M University-Corpus Christi (TAMU-CC).
**Data accessibility**	Repository Name: LagunaMadreWaterAirTempCleaner [4] Data Identification Number: 10.5281/zenodo.10064703 GitHub Repository URL: conrad-blucher-institute/LagunaMadreWaterAirTempDataCleaner (github.com)

## 1 Value of the Data

• The data described in this article can be used to (1) analyze daily, seasonal, and interannual variability of air and water temperature in Laguna Madre, TX, (2) study the relationship between air and water temperatures, (3) forecast or analyze trends in air and water temperatures, (4) reduce uncertainty in air and water temperature forecasts, and (5) enhance water and natural resource and risk management decisions during freeze or drought events.
• The most significant contribution of this paper is the creation of a complete 10-year time-series dataset. A minimal gap (<2%) dataset is highly valuable for the calibration of Artificial Intelligence (AI) models.
• This dataset can be valuable to data scientists, natural and water resource managers, climate scientists, forecasters, and others who are in need of reliable air and water temperature data.
• The imputed dataset provides reliable air and water temperature information in one of the most important development areas for juvenile endangered green sea turtles in the western Gulf of Mexico.

## 2 Data Description

The dataset presented in this article is representative of hydrological and atmospheric conditions within the Laguna Madre TX, a shallow estuarine system located in southern Texas. Water temperatures can change very rapidly in the Laguna Madre because of the cooling air temperatures brought in by cold fronts but also because of the hydrodynamics of the Laguna Madre itself (e.g., wind-driven and well-mixed, shallow, restricted flow from the Gulf of Mexico [GoM]). Given the climatic conditions of the area, the lagoon system is sometimes susceptible to freezing air temperatures when cold fronts travel toward the coast during the cold season, impacting water temperatures [9]. Climatic and oceanic factors such as air temperature, sea surface temperature, barometric pressure, wind direction, and wind speed influence cold-stunning events along the Texas coast[9]. However, Tissot et al. showed that air temperature was by far the main forcing on water temperatures in the Laguna Madre (with the exception of waters by deep draft ship channels, e.g., Brownsville ship channel) [10][10]. Cold fronts can substantially lower air temperatures by more than 10°C in less than 24 hours[9], significantly decreasing water temperature in the Laguna Madre [10]. These conditions can cause threatened green sea turtles and other marine life to become “cold-stunned,” no longer capable of moving or protecting themselves.

The dataset described in this article consists of 10 years of air and water temperature measurements from 2012 to 2022 extracted from the Texas Coastal Ocean Observation Network (TCOON) [7], initially used to forecast water temperatures in the area of interest. TCOON has been noted as a valuable hydrological/environmental data retrieval tool since 1991 for the state of Texas, collecting water level, wind speed, barometric pressure, salinity, water quality, and other environmental data along several locations along the Texas coast [8]. TCOON has been utilized by the National Ocean and Atmospheric Administration (NOAA), US Army Corps of Engineers (USACE), and the Conrad Blucher Institute (CBI) for many applications, resulting in many benefits to the agencies (e.g., Texas General Land Office, Texas Water Development Board) and communities that each TCOON station serves. However, the maintenance of TCOON was temporarily halted starting in 2014 for one or more years, depending on location, before resuming data collection. However, the 2014 halt, occasional extreme events, data transmission problems and harshness of the coastal environment led to the reduction of data quality, leading to large gaps of missing data and at times erroneous data. The reduction in the data quality along the Texas coast has limited the usability and reliability of the data for a diverse set of users. This paper focuses on enhancing the usability of air temperature (ATP) and water temperature (WTP) data acquired from TCOON by combining statistical processing and utilizing highly correlated locations (depending on the variable and location; Table 1). The goal is to improve its applicability for diverse analysis and forecasting models, aiming to restore its value in scientific research, analysis, and various management decision-making processes.

**Table 1:** Pearson correlation coefficients (%) of (A) air and (B) water temperature measurements (°C) of various stations located in the Upper Laguna Madre, including South Bird Island (SBI), Packery Channel, Baffin Bay (BB), and National Park Service (NPS)-SBI stations.

*(A)*	**Air Temperature**			*(B)*	**Water Temperature**	
	**SBI**	**Packery**	**BB**		**SBI**	**NPS-SBI**
**SBI**	100%	99.39%	99.30%	**NPS-SBI**	100%	99.37%
**Packery**	99.39%	100%	99.20%	**SBI**	99.37%	100%
**BB**	99.30%	99.20%	100%			

## 3 Experimental Design, Materials, and Methods

### 3.1 Study location - Laguna Madre estuarine system

**Fig. 1:**
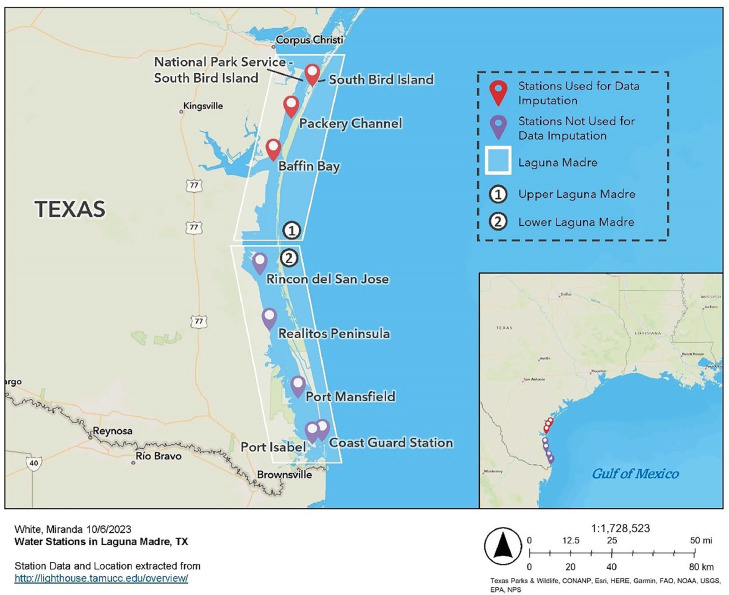
Map of water stations located in Laguna Madre, TX. Stations that were used for the imputation process are labeled in red, while the remaining stations that are not used for the newly gap-filled dataset are labeled in purple.

The Laguna Madre is characterized as a shallow (≃1.2 m [1]) estuarine system that is divided into two sections: the upper and lower Laguna Madre. Both sections cover approximately 1133 km^2^ [5], separated by an extensive area of wind tidal flats and hydrologically connected by the Gulf Intracoastal Waterway (GIWW) also known as the “Land Cut”. The estuarine system has highly restricted flows in and out of the GoM with only three outlets that allow for water transfer from the bay to the Gulf: Brazos Santiago Pass, Mansfield Channel, and Packery Channel [9]. Both sections of Laguna Madre also have minimal freshwater inflow, historically often expressing a negative freshwater inflow balance [12]. Because of this, the system is known to be one of the six most hypersaline lagoons in the world, with salinity levels ranging from 26 to 50 g/kg depending on local rainfall [[9], [12]]. During the passage of cold fronts, water temperatures in Laguna Madre are driven by generally homogeneous air temperatures brought in by cold fronts and can be considered homogeneous as well [9]. Despite these harsh saline conditions and occasional extreme cold events, the Laguna Madre is an extremely productive bay system, home to numerous commercially and ecologically valuable marine species. There are approximately 9 present and historical TCOON and National Park Service (NPS) stations placed within the Laguna Madre system (Fig. 1).

### 3.2 Data acquisition

Hourly air and water temperature time-series data from TCOON and NPS stations within the upper Laguna Madre were acquired (lighthouse.tamucc.edu). The selected locations are South Bird Island, Packery Channel, Baffin Bay, and NPS-South Bird Island [NPS-SBI] stations. The data acquired from the multiple stations were analyzed to assess the variability and heterogeneity of water and air temperatures between each station in order to understand the range of suitability of the nearby stations for potential data imputation.

### 3.3 Percentage of missing data

The unprocessed 2010–2022 air and water temperature dataset from all stations contained substantial proportions of missing data (Table 2). Within the initial acquired data, data prior to 2012 had more than 90% missing data and therefore was excluded.

**Table 2:** Percentages (%) of missing values for the original datasets of the South Bird Island (SBI), Packery Channel, Baffin Bay (BB), and National Park Service-South Bird Island (NPS-SBI) stations per year.

**Year**	**ATP**			**WTP**	
	**SBI (%)**	**Packery (%)**	**BB (%)**	**SBI (%)**	**NPS-SBI (%)**
**2012**	0.17	0.02	12.9	0.17	5.05
**2013**	0.09	0.74	0.06	1.26	9.92
**2014**	3.93	0.01	0.08	3.93	7.72
**2015**	30.1	0.05	0.30	30.1	0.73
**2016**	25.9	0.03	0.48	3.01	0.09
**2017**	26.4	0.31	0.96	26.3	0.67
**2018**	2.23	4.50	3.00	2.17	9.10
**2019**	19.6	1.26	1.96	28.4	0.08
**2020**	64.1	10.4	4.02	73.6	0.24
**2021**	5.76	18.6	20.2	5.76	0.11
**2022**	3.36	0.48	0.49	3.34	1.00

### 3.4 Experimental design

The primary objective is to create a dataset that is representative of the upper Laguna Madre with minimal gaps (<2%) for each year within the time-series dataset. Therefore, each station used for experimentation for the data imputation method was analyzed using Pearson correlations between each combination. It was observed that each station combination for both air and water temperatures had correlation values higher than 99% (Table 1). This justifies the use of the selected stations for use in our proposed data imputation methods. After data imputation methods were applied and the final missing percentages were computed for each combination, the imputed dataset that contained the lowest percentage of missing data was selected for each of the two variables. All imputation and evaluation methods were implemented with the Python programming language.

### 3.5 Gap-filling methods

Two different processes were used to gap-fill missing data within the 2012–2022 air and water temperature dataset, dependent on the length of the gap of missing data. With this in mind, the gaps were classified as short and long gaps. Short and long gaps for missing air and water temperatures were defined by the dynamics of the local physical conditions of the Laguna Madre system. Short gaps were characterized as gaps that were less than or equal to 3 hours for air temperature and 5 hours for water temperature. Any remaining gaps that were larger than the defined short gaps were defined as long gaps.

**Fig. 2:**
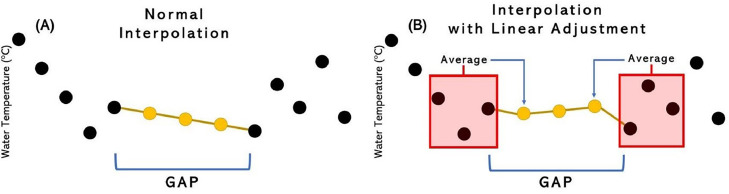
(A) Normal interpolation method versus (B) interpolation method using short gap method with linear adjustment.

*Short-Gap Interpolation Method:* Gap-filling methods utilized for short gaps involved linear interpolation methods. To interpolate the small gaps, the averages of the last three measurements before and after the gap were computed. The two computed averages were used as the first and the last interpolated values within the gap. Rather than using the gap's first and last measurements, the average of the previous and next three values added robustness to the interpolation approach (Fig. 2).

Although this approach is viable for a majority of the dataset, this gap-filling method was not found to be suitable for extreme cold events, where water and air temperatures drop significantly very rapidly [9]. Studies show that air temperatures in the area can drop by more than 10°C in less than 24 hours [[2], [9]]. To ensure that the proposed approach would not fail in these scenarios, the approach was applied when the following conditions were met: (1) the range of the three values before the beginning of the gap and the range of the value after the end of the gap is smaller than 1.5°C; (2) the absolute difference between the mean values before and the after the gaps is smaller than 1.5°C. If these conditions were not met, then the short gaps were not filled with our proposed method.

*Long-Gap Imputation Method:* Once short-gap interpolation methods were applied to all selected stations, long-gap imputation methods were implemented based on all combinations above (Table 2). The stations where gap-filling was applied are referenced as the main stations, and the stations that were used to gap-fill are referenced as the nearby stations. When analyzing the data, it was observed that some of the long gaps in the dataset extended over multiple days, making linear interpolation approaches unreliable for addressing these cases. Long gaps of missing data within the main datasets were thus filled with the measurements of the selected nearby stations after linear adjustments of bias of the start and end of the gaps were accounted for.

To apply the linear adjustment used for the long gap-filling process, the averages of the last three measurements before the start of the gap and the first three measurements after the end of the gap were computed for both the main and nearby stations. The difference between the average measurement of the main station and that of the nearby station was then calculated and extracted. The corrected value was obtained by averaging the differences between station measurements before and after the gap. This corrected value was added to the nearby station measurement to obtain the value used to fill the missing measurement. Similar to what was observed when using the proposed short-gap interpolation approach, the long-gap imputation approach worked for most cases, however it was observed that the method failed when sudden changes in temperatures occurred. To ensure that the proposed approach would not fail in these scenarios, the approach was applied when the following conditions were met: the difference between the average of the three values for both the original and nearby station before the beginning and after the end of the gap was smaller than 1.5°C.

Linear interpolation methods that were utilized for short gaps were then facilitated again after long gaps were filled to account for new short gaps that formed after data substitution processes were completed. Final missing data percentages after data imputation methods: Once data imputation methods were complete, final percentages of missing air temperature data showed that the utilization of the Packery Channel dataset as the main station in combination with Baffin Bay resulted in the lowest percentage of missing air temperature data (Table 3). It was also determined that the utilization of the NPS-SBI dataset as the main station in combination with the South Bird Island station resulted in the lowest percentage of missing water temperature data (Table 3). Thus, the final dataset is comprised of air and water temperature measurements from Packery and NPS-SBI stations, respectively. In the case of the Packery measurements, the dataset was filled using Baffin Bay, whereas the NPS-SBI measurements were filled using the SBI station. The dataset included only the years that contained less than 2% of missing data after completion of the gap-filling process. All years from 2012 to 2022 contained less than 1.2% of missing data with the exception of 2021 (15.5% of missing data; Table 3), therefore was excluded from the final dataset (Table 3).

**Table 3:** Percentage of missing values before and after the long gap-filling method has been applied for all station combinations. The main stations are listed first, and the nearby station used for gap-filling is labeled by and succeeds the dash (-).

**Year**	**ATP**						**WTP**	
	**SBI-Packery**	**SBI- BB**	**Packery- SBI**	**Packery- BB**	**BB- SBI**	**BB-Packery**	**SBI- NPS-SBI**	**NPS-SBI- SBI**
**2012**	0.01	0.05	0.00	0.00	12.3	12.3	0.01	1.12
**2013**	0.05	0.05	0.05	0.05	0.05	0.05	1.18	0.19
**2014**	2.69	2.71	0.00	0.00	0.30	0.03	2.71	1.30
**2015**	5.73	26.4	0.02	0.00	0.10	0.02	5.67	0.49
**2016**	2.11	1.98	0.01	0.01	0.33	0.20	1.20	0.00
**2017**	18.2	18.2	0.15	0.05	0.74	0.73	18.3	0.39
**2018**	1.79	1.79	0.66	0.92	1.82	0.95	1.79	0.63
**2019**	16.9	15.2	0.19	0.19	1.83	0.19	22.6	0.08
**2020**	27.6	21.7	10.2	0.05	3.92	3.89	27.6	0.07
**2021**	2.57	2.01	16.5	15.5	18.3	19.9	2.59	0.01
**2022**	0.84	0.82	0.31	0.29	0.30	0.30	0.84	0.40

Table 4 shows the significant effect that the gap-filling approach had on the original datasets. In the original datasets, only 5 years had both water and air temperature data with less than 2% of missing values. However, after the use of the imputation methods, all years except for 2021 accomplished this goal, resulting in 10 years of data with less than 2% missing data (Table 4). This is a significant improvement for water and air temperature data within the Laguna Madre and extremely valuable for the application of artificial intelligence (AI) and machine learning (ML) modeling particularly when continuous time-series inputs are necessary such as for long short-term memory model [3], recurrent neural networks [6], and transformer architectures [11].

**Table 4:** Percentage (%) of missing values for (1) the original datasets before imputation methods were employed and (2) the final datasets after imputation methods were employed.

**Year**	**Packery ATP**		**NPS-SBI WTP**	
	**Original**	**Final**	**Original**	**Final**
**2012**	0.02	0.00	5.05	1.12
**2013**	0.74	0.05	9.92	0.19
**2014**	0.01	0.00	7.72	1.30
**2015**	0.05	0.00	0.73	0.49
**2016**	0.03	0.01	0.09	0.00
**2017**	0.31	0.05	0.67	0.39
**2018**	4.50	0.92	9.10	0.63
**2019**	1.26	0.19	0.08	0.08
**2020**	10.4	0.05	0.24	0.07
**2021**	18.6	15.5	0.11	0.01
**2022**	0.48	0.29	1.00	0.40

### 3.6 Evaluation of gap-filling method

In order to evaluate the proposed data imputation method, both NPS-SBI and Packery Channel datasets were used to assess the reliability of the methods. Random artificial gaps were created, representing up to 10% of the dataset size for each year. These gaps were then filled utilizing the proposed methods and evaluated using various metrics (e.g. mean absolute error [MAE] (Eq. 4), root mean squared error [RMSE] (Eq. 3), maximum 10% error [ME10] (Eq. 6)) to determine the reliability and validity of the method. The short and long-gap imputation methods were evaluated separately. For the short gap interpolation evaluation, 3-hour gaps were created for ATP measurements and 5-hour gaps were created for =WTPmeasurements. Random placement of these gaps was conducted for each year and variable. This assessment created gaps of maximum length of the short gaps for both ATP and WTP. This means that in the case of the use or observation of smaller gaps than the defined maximum length within the small-gap interpolation method, the interpolation evaluation results would be slightly better. For the long gap imputation evaluation, gaps ranging from 6 to 168 hours were randomly created, both in length and placement, within the WTP and ATP time series. This range is representative of 95% of the long gaps observed within the original dataset and was used to ensure a broad representation of the potential missing value scenarios. Both evaluation methods were applied thirty times in order to capture the variability of the observed errors (e.g., mean ± standard deviation) that were computed using the metrics noted and defined below:

(1) $$ME=\;\frac{1}{n}\sum_{i=1}^{n}\left(x_{i}-x\right)$$

(2) $$MSE=\;\frac{1}{n}\sum_{i=1}^{n}{\left(x_{i}-x\right)}^{2}$$

(3) $$RMSE=\;\sqrt{\frac{\sum_{i=1}^{n}{\left(x_{i}-x\right)}^{2}}{n}}$$

(4) $$MAE=\;\frac{1}{n}\sum_{i=1}^{n}\left|x_{i}-x\right|$$

(5) $$Max\left|E\right|=max\left(\left|x_{i_{1}}-x_{1}\right|,\left|x_{i_{1}}-x_{1}\right|,\ldots ,\;\left|x_{i_{n}}-x_{n}\right|\right)$$

(6) $$Max10\%\left(MAE\right)=\;\sum_{i=1}^{n}max10\%\left(MAE\;sorted\;residuals\right)$$

Where *x_i_* is the observed values, *x* is the interpolated values, and *n* is the number of data points.

Results for the short gap interpolation method for the 30 trials show that ATP MAE values (Eq. 4) for all years were below 0.50°C, while the maximum 10% mean error (Max10%(MAE)) (Eq. 6) averaged 1.12 ± 0.03°C (Table 5) for all years. WTP results for the short gap interpolation evaluation show similar results for MAE, displaying MAE values below 0.50 °C and Max10%(MAE) values no higher than 1.40°C (Table 6) for the full WTP dataset.

**Table 5:** Evaluation metrics for the short gap-filling approach for Packery Channel ATP measurements (i.e., mean ± standard deviation of 30 trial runs).

**Year**	**ME (°C)**	**MSE (°C)**	**RMSE (°C)**	**MAE (°C)**	**Max|E| (°C)**	**Max10%(MAE) (°C)**
**2012**	0.02 ± 0.04	0.24 ± 0.03	0.49 ± 0.03	0.35 ± 0.02	2.53 ± 0.91	1.11 ± 0.09
**2013**	0.01 ± 0.03	0.23 ± 0.04	0.48 ± 0.05	0.33 ± 0.02	2.79 ± 0.79	1.10 ± 0.12
**2014**	0.01 ± 0.03	0.25 ± 0.04	0.50 ± 0.04	0.36 ± 0.03	2.40 ± 0.57	1.15 ± 0.11
**2015**	0.01 ± 0.03	0.25 ± 0.04	0.50 ± 0.04	0.35 ± 0.02	2.87 ± 0.85	1.15 ± 0.11
**2016**	0.00 ± 0.03	0.24 ± 0.04	0.49 ± 0.04	0.34 ± 0.02	2.48 ± 0.72	1.14 ± 0.09
**2017**	0.02 ± 0.04	0.25 ± 0.05	0.50 ± 0.05	0.34 ± 0.02	2.69 ± 0.90	1.14 ± 0.12
**2018**	0.00 ± 0.03	0.23 ± 0.04	0.47 ± 0.04	0.33 ± 0.02	2.56 ± 0.79	1.09 ± 0.10
**2019**	0.00 ± 0.03	0.25 ± 0.04	0.50 ± 0.04	0.35 ± 0.02	2.93 ± 0.84	1.15 ± 0.11
**2020**	0.01 ± 0.03	0.24 ± 0.04	0.49 ± 0.04	0.34 ± 0.02	2.35 ± 0.47	1.13 ± 0.11
**2021**	0.00 ± 0.04	0.23 ± 0.04	0.48 ± 0.04	0.34 ± 0.02	2.41 ± 0.63	1.09 ± 0.11
**2022**	0.01 ± 0.03	0.22 ± 0.04	0.47 ± 0.04	0.33 ± 0.02	2.63 ± 1.12	1.07 ± 0.10

**Table 6:** Evaluation metrics for the short gap-filling approach for NPS-SBI WTP measurements (i.e., mean ± standard deviation of 30 trial runs).

**Year**	**ME (°C)**	**MSE (°C)**	**RMSE (°C)**	**MAE (°C)**	**Max|E| (°C)**	**Max10%(MAE) (°C)**
**2012**	0.04 ± 0.06	0.38 ± 0.04	0.62 ± 0.03	0.47 ± 0.03	2.03 ± 0.23	1.33 ± 0.07
**2013**	0.04 ± 0.03	0.30 ± 0.03	0.55 ± 0.03	0.41 ± 0.02	1.93 ± 0.42	1.19 ± 0.06
**2014**	0.04 ± 0.03	0.32 ± 0.03	0.57 ± 0.03	0.42 ± 0.02	2.15 ± 0.41	1.25 ± 0.07
**2015**	0.05 ± 0.03	0.28 ± 0.03	0.53 ± 0.03	0.38 ± 0.02	1.92 ± 0.13	1.22 ± 0.07
**2016**	0.05 ± 0.03	0.29 ± 0.03	0.53 ± 0.03	0.40 ± 0.03	1.94 ± 0.35	1.16 ± 0.06
**2017**	0.06 ± 0.04	0.33 ± 0.03	0.57 ± 0.03	0.43 ± 0.02	2.07 ± 0.30	1.25 ± 0.06
**2018**	0.04 ± 0.03	0.31 ± 0.04	0.55 ± 0.03	0.41 ± 0.03	1.82 ± 0.26	1.23 ± 0.08
**2019**	0.02 ± 0.03	0.27 ± 0.03	0.52 ± 0.03	0.39 ± 0.03	1.97 ± 0.37	1.14 ± 0.06
**2020**	0.03 ± 0.02	0.27 ± 0.03	0.52 ± 0.02	0.39 ± 0.02	1.79 ± 0.20	1.14 ± 0.05
**2021**	0.04 ± 0.04	0.28 ± 0.02	0.53 ± 0.02	0.40 ± 0.02	1.90 ± 0.30	1.14 ± 0.05
**2022**	0.03 ± 0.04	0.32 ± 0.04	0.57 ± 0.03	0.42 ± 0.02	2.08 ± 0.35	1.25 ± 0.07

Results for the long gap imputation method for the 30 trial runs show that ATP MAE values averaged 0.87 ± 0.14°C for the full ATP dataset (Table 7). Max10%(MAE) values averaged 2.63 ± 0.34°C for all years=(Table 8). WTP results for the long gap interpolation method reflected MAE values that averaged 0.88 ± 0.69°C for the full WTP dataset (Table 8). Max10%(MAE) averaged to 2.99 ± 1.51°C for all years (Table 8). These results justify the application of the proposed data imputation approach.

**Table 7:** Evaluation metrics for the long gap-filling approach for Packery Channel ATP measurements when using Packery Channel as the main station and Baffin Bay as the adjacent station (i.e., mean ± standard deviation of 30 trial runs).

**Year**	**ME (°C)**	**MSE (°C)**	**RMSE (°C)**	**MAE (°C)**	**Max|E| (°C)**	**Max10%(MAE) (°C)**
**2012**	0.39 ± 0.08	1.16 ± 0.90	1.03 ± 0.32	0.69 ± 0.10	7.99 ± 2.59	2.30 ± 0.78
**2013**	0.23 ± 0.07	1.00 ± 0.63	0.98 ± 0.22	0.70 ± 0.06	7.81 ± 2.29	2.23 ± 0.52
**2014**	0.48 ± 0.08	1.13 ± 0.36	1.05 ± 0.14	0.78 ± 0.06	7.71 ± 2.26	2.33 ± 0.33
**2015**	0.44 ± 0.08	1.28 ± 0.54	1.11 ± 0.22	0.77 ± 0.08	8.16 ± 2.99	2.50 ± 0.53
**2016**	0.48 ± 0.08	1.25 ± 0.46	1.11 ± 0.18	0.78 ± 0.07	7.82 ± 2.97	2.49 ± 0.50
**2017**	0.51 ± 0.10	1.25 ± 0.21	1.11 ± 0.09	0.82 ± 0.07	6.21 ± 0.99	2.53 ± 0.24
**2018**	0.61 ± 0.08	1.37 ± 0.53	1.15 ± 0.19	0.85 ± 0.07	8.57 ± 3.54	2.53 ± 0.38
**2019**	0.77 ± 0.08	1.87 ± 0.88	1.34 ± 0.26	1.00 ± 0.09	10.75 ± 2.88	2.86 ± 0.65
**2020**	0.75 ± 0.10	1.74 ± 0.34	1.31 ± 0.13	0.99 ± 0.08	7.71 ± 0.95	2.95 ± 0.36
**2021**	0.73 ± 0.14	2.35 ± 0.75	1.51 ± 0.25	1.07 ± 0.10	10.59 ± 4.01	3.37 ± 0.68
**2022**	0.91 ± 0.12	1.89 ± 0.77	1.36 ± 0.22	1.07 ± 0.08	7.98 ± 3.13	2.83 ± 0.53

**Table 8:** Evaluation metrics for the long gap-filling approach for WTP measurements when using NPS-SBI as the main station and SBI as the adjacent station (i.e., mean ± standard deviation of 30 trial runs).

**Year**	**ME (°C)**	**MSE (°C)**	**RMSE (°C)**	**MAE (°C)**	**Max|E| (°C)**	**Max10%(MAE) (°C)**
**2012**	−2.30 ± 0.05	5.68 ± 0.23	2.38 ± 0.05	2.30 ± 0.05	4.48 ± 0.26	3.42 ± 0.13
**2013**	−1.82 ± 0.09	4.31 ± 0.70	2.07 ± 0.16	1.87 ± 0.07	7.40 ± 3.58	3.30 ± 0.44
**2014**	−0.04 ± 0.05	0.28 ± 0.11	0.53 ± 0.09	0.36 ± 0.03	3.58 ± 1.68	1.21 ± 0.21
**2015**	−0.50 ± 0.16	2.26 ± 0.77	1.48 ± 0.25	0.84 ± 0.11	8.46 ± 2.13	3.58 ± 0.68
**2016**	−0.08 ± 0.05	0.45 ± 0.44	0.61 ± 0.29	0.26 ± 0.05	7.68 ± 3.50	1.16 ± 0.48
**2017**	0.12 ± 0.25	3.39 ± 2.36	1.74 ± 0.62	0.78 ± 0.23	10.10 ± 3.23	4.71 ± 1.72
**2018**	−0.08 ± 0.07	0.46 ± 0.32	0.65 ± 0.21	0.31 ± 0.06	5.81 ± 2.80	1.55 ± 0.50
**2019**	−0.08 ± 0.10	0.80 ± 0.78	0.81 ± 0.39	0.41 ± 0.11	6.14 ± 3.13	1.89 ± 0.93
**2020**	−0.09 ± 0.32	1.83 ± 2.47	1.08 ± 0.83	0.52 ± 0.37	5.51 ± 3.53	2.74 ± 2.32
**2021**	−0.94 ± 0.22	7.26 ± 2.67	2.66 ± 0.47	1.49 ± 0.21	11.90 ± 4.20	6.09 ± 1.28
**2022**	0.01 ± 0.17	1.91 ± 1.15	1.32 ± 0.42	0.62 ± 0.15	10.14 ± 3.51	3.28 ± 1.10

## Limitations

One limitation of the proposed imputation method is the need for highly correlated nearby stations to apply the long gap-filling approach. If the nearby stations did not exist or the nearby station data was not of good quality during the main station gaps, then the long gap-filling approach could not be applied. Another limitation is that the proposed gap-filling approach cannot be applied when the missing data corresponds to extreme events.

## Ethics Statement

The current work meets the ethical requirements for publication in Data in Brief and does not involve human subjects, animal experiments, or any data collected from social media platforms.

## CRediT authorship contribution statement

**Miranda C. White:** Conceptualization, Methodology, Writing – review & editing. **Marina Vicens-Miquel:** Conceptualization, Methodology, Writing – review & editing. **Philippe Tissot:** Supervision, Data curation, Methodology, Writing – review & editing. **Evan Krell:** Project administration.
